# The Anticoagulant Effect of PGI2S and tPA in Transgenic Umbilical Vein Endothelial Cells Is Linked to Up-Regulation of PKA and PKC

**DOI:** 10.3390/ijms15022826

**Published:** 2014-02-19

**Authors:** Jian-Hua Wang, Lin-Jing Yuan, Zhi-Min Zhong, Zhe-Sheng Wen, Jian-Ming Deng, Rong-Xin Liang, Min Zheng

**Affiliations:** 1 Cardiovascular Surgery Department, Second People’s Hospital of Guangdong Province, 1 Shi-liu Gang Road, Guangzhou 510317, China; E-Mails: zmin908@sina.com (Z.-M.Z.); shiliyingsly@sina.com (R.-X.L.); 2 State Key Laboratory of Oncology in Southern China, Sun Yat-sen University Cancer Center, 651 Dongfeng Road East, Guangzhou 510060, China; E-Mail: yuanlj@sysucc.org.cn; 3 Department of Gynecology, Sun Yat-sen University Cancer Center, 651 Dongfeng Road East, Guangzhou 510060, China; 4 Department of Chest, Sun Yat-sen University Cancer Center, 651 Dongfeng Road East, Guangzhou 510060, China; E-Mail: wenzhsh@sysucc.org.cn; 5 Department of Chest, Second People’s Hospital of Guangdong Province, 1 Shi-liu Gang Road, Guangzhou 510317, China; E-Mail: DoctorDengjianming@126.com

**Keywords:** anticoagulant, coronary artery bypass graft, endothelial cells, *PGI2S*, *tPA*

## Abstract

The selection of vascular grafts for coronary artery bypass surgery is crucial for a positive outcome. This study aimed to establish a novel line of vascular endothelial cells with a potent anticoagulant effect. A lentiviral vector was used to stably transfect human umbilical vein endothelial cells (HUVECs) with *PGI2S* alone (HUVEC-*PGI2S*) or both *PGI2S* and *tPA* (HUVEC-*PGI2S-tPA*). Both HUVEC-PGI2S and HUVEC-*PGI2S-tPA* cells over-expressing *PGI2S* and *tPA* were compared to mock-transfected cells. The enzyme-linked immuno sorbent assay (ELISAs) demonstrated that the anticoagulation components, *ATIII* and *PLG*, were up-regulated and coagulation factor *FVIII* was down-regulated in both cell lines. QRT-PCR and western blotting demonstrated the vasodilation and platelet disaggregation proteins *PKA*, *PKC*, and *PTGIR* were up-regulated in both cell lines, but *MAPK* expression was not altered in either cell line. However, cell viability and colony formation assays and cell cycle analysis demonstrated that both cell lines had a lower rate of cell growth and induced G1 phase arrest. HUVEC-*PGI2S* and HUVEC-*PGI2S-tPA* cells have a potent anticoagulant effect and their use in vascular heterografts may decrease the risk of thrombosis.

## 1. Introduction

Since the first successful coronary artery bypass operation was performed by Goetz *et al.* in 1960 [1,2], coronary artery bypass surgery, also known as coronary artery bypass graft (CABG) surgery, has become the most common surgical method of treating coronary heart disease [3]. The type of conduit used for the bypass is crucial for a positive outcome, and affects the subsequent quality of life of the patient. Currently, four main classes of coronary artery bypass conduits are used: veins or arteries from autografts, allografts, heterografts, or artificial blood vessels [4,5]. Vascular grafts using autologous blood vessels are not ideal, due to the damage that the operation can cause and the potential for formation of vascular graft lesions. Allograft conduits have limited sources and introduce the problem of rejection by the recipient. Artificial blood vessels, made of endothelial cells adhering to the inner wall of an artificial blood vessel, do not always meet the physiological criteria required for transplantation due to the unsuitability of the materials used. Currently, heterografts are the most favorable option, as they possess physically suitable structures and are readily available. However, unprocessed heterografts can induce a strong rejection reaction in the recipient. Therefore, replacement of the epithelial cells with cells that have lower immunogenicity and stronger anticoagulation effect may enable the development of more suitable heterografts as conduits for CABG.

Vascular endothelial cells play a role in immunological rejection and the coagulation process, both of in which can lead to thrombosis and ultimately even graft failure. Endothelial cell disorders can result in blood clots, as the vascular endothelium forms a barrier between the blood and the vascular wall that isolates antigens from the host immune system, and the coagulation system from the coagulation cascade promoter. Vascular endothelial cells can also generate anticoagulation components [6]. In 1979, Herring *et al.* [7,8] implanted endothelial cells into artificial blood vessels, and then transferred these vessels into the arterial system of dogs. With the implanted endothelial cells in the blood vessels, the postoperative non-thrombotic area reached 70%, compared to 20% in vessels without implanted cells [7,8]. Other reports have confirmed that seeding artificial blood vessels with endothelial cells can significantly improve the patency rates of grafts, and also reduce platelet aggregation [9,10]. Thrombosis is the most common complication of CABG, and leads to vascular clogging and even graft failure [6,11]. Therefore, the use of artificially-modified epithelial cells with reduced immunogenicity and increased anticoagulant activity to replace the endothelium of a vascular allograft or heterograft could produce a novel type of conduit that does not share the limitations of the options currently available [10,12].

The fibrinolytic system and clotting system operate a system of checks and balances to ensure that thrombosis functions normally. It is possible that artificially increasing the expression of anticoagulants and decreasing the expression of proteins involved in fibrinolysis in endothelial cells may improve their use in artificial vascular grafts. Some reports have shown that when the *tissue plasminogen activator* (*tPA*) gene, a promoter of the fibrinolytic system which acts to prevent thrombosis, is transfected into aortic endothelial cells, expression of *tPA* in the blood remains elevated for several hours, and can effectively prevent formation of a thrombosis in a graft. Dicheck *et al.* reported that intravascular stents seeded with *tPA*-transfected vascular endothelial cells could secrete *tPA in vitro* and prevent coagulation induced by stents [13]. Surface-retained t*PA* on vascular endothelial cells has also been show to be essential for effective fibrinolysis on vascular endothelial cells [14,15].

Prostacyclin *(PGI2*) is an important inhibitor of platelet activation, and can inhibit formation of the platelet plug during primary hemostasis, and also acts as a vasodilator to reduce the clotting process. PGI2 is predominantly synthesized in vascular endothelial cells from prostaglandin H2 by *PGI2 Synthase* (*PGI2S*; also called *prostaglandin-endoperoxide synthase 2*), a member of the cytochrome P450 isomerase family [16–18]. Some reports have shown that mutations in the *PGI2S* gene increase the risk of cardiovascular disease [19,20]. Aspirin is often commonly administered after *CABG*, and is thought to inhibit platelet function by inhibiting *prostaglandin-endoperoxide synthase 1* (*COX-1*), and the resulting inhibition of *PGI2* synthesis is also thought to disturb the balance of the coagulation system [3]. Therefore, *PGI2S* may represent a vital component in the creation of endothelial cells with enhanced anticoagulant activity. It may be possible to produce hybrid endothelial cells with increased anticoagulant activity by transfecting endothelial cells with other potent anticoagulant factors: either *PGI2S* on its own or in combination with *tPA*. These hybrid cells may have the potential to provide an ideal source of endothelial cells for vascular grafts.

In this study, we used human umbilical vein endothelial cells (HUVECs), an endothelial cell line with relatively low immunogenicity [21,22]. We established two stable HUVEC cell lines: the first, HUVEC-*PGI2S*, was transfected solely with the *PGI2S* gene, and the second, HUVEC-*PGI2S-tPA*, was transfected with both the *PGI2S* and *tPA* genes, to explore any possible synergistic anticoagulant effect. The effects of over-expressing *PGI2S* and *tPA* on cell proliferation were examined. Furthermore, the effects of over-expressing *PGI2S* and *tPA* on several anticoagulant signaling pathway proteins and the expression levels of anticoagulant and coagulation factors were examined, in order to investigate the potential anticoagulant properties of these cells.

## 2. Results

### 2.1. PGI2S and tPA Are Over-Expressed in HUVEC-PGI2S and HUVEC-PGI2S-tPA Cell Lines

After cloning the *PGI2S* and *tPA* cDNAs from the human liver cell line L-02, the lentiviral backbone vector, pSin-EF2-puro-oligo was utilized to construct HUVEC cell lines stably over-expressing *PGI2S* (HUVEC-*PGI2S*), or *PGI2S* and *tPA* (HUVEC-*PGI2S-tPA*). A negative control cell line, HUVEC-mock, was created using the empty lentivirus backbone vector.

**Figure 1.**
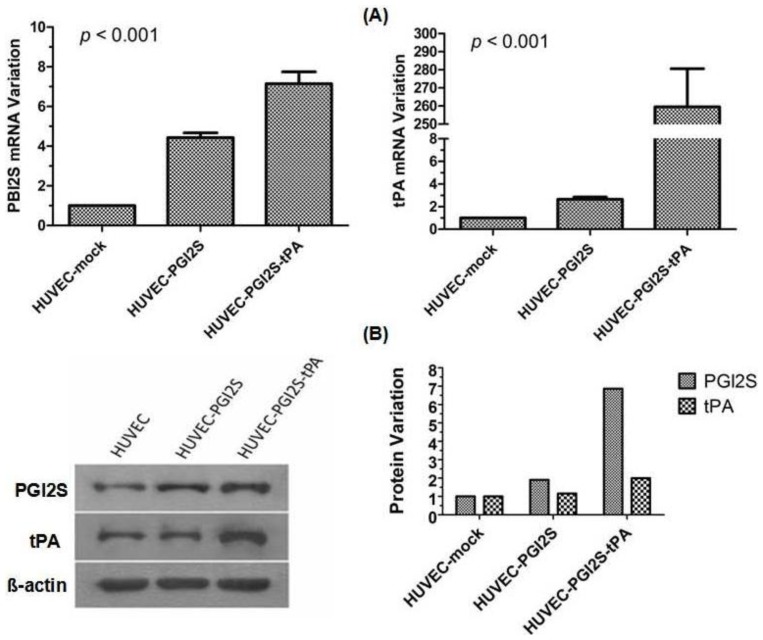
Expression of *PGI2S* and *tPA* in HUVEC-mock, HUVEC-*PGI2S*, and HUVEC-*PGI2S-tPA* cell lines. (**A**) *PGI2S* and *tPA* mRNA expression were analyzed by quantitative real-time PCR and expressed relative to the HUVEC-mock cell line. Values are means ± S.D. of triplicates. *p* values were calculated using one-way ANOVA; (**B**) Analysis of *PGI2S* and *tPA* protein expression by Western blotting in HUVEC-mock, HUVEC-*PGI2S*, and HUVEC-*PGI2S-tPA* cell lines. The Western blotting analyses were performed in triplicate.

The mRNA and protein expression of *PGI2S* and *tPA* were analyzed by qRT-PCR and Western blotting. Compared to HUVEC-mock cells, *PGI2S* mRNA expression was approximately 4.4-fold higher in HUVEC-*PGI2S* cells, and approximately 7.1-fold higher when simultaneously over-expressed with *tPA* in HUVEC-*PGI2S-tPA* cells (*p* < 0.001); *tPA* mRNA expression was approximately 2.6-fold and 259.5-fold higher in HUVEC-*PGI2S* and HUVEC-*PGI2S-tPA* cells, respectively, compared to HUVEC-mock cells (*p* < 0.001; Figure 1A). These increases in mRNA expression were matched by similar increases in *PGI2S* and *tPA* protein expression. Densitometry revealed that the protein expression levels of *PGI2S* were 1.9-fold higher in HUVEC-*PGI2S* cells and 6.9-fold higher in HUVEC-*PGI2S-tPA* cells compared to HUVEC-mock cells. The protein expression levels of *tPA* were not increased in HUVEC-*PGI2S* cells but were 2.0-fold higher in HUVEC-*PGI2S-tPA* cells compared to HUVEC-mock cells (Figure 1B).

### 2.2. Cell Growth Is Restricted in HUVEC-PGI2S and HUVEC-PGI2S-tPA Cell Lines

**Figure 2.**
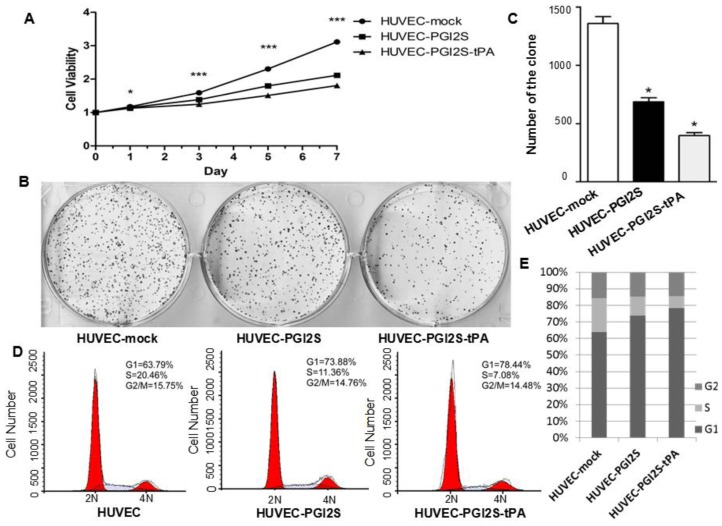
Cell growth and colony formation ability of HUVEC-mock, HUVEC-P*GI2S* and HUVEC-*PGI2S-tPA* cell lines. (**A**) Cell viability of HUVEC-*PGI2S* and HUVEC-*PGI2S-tPA* cells determined by the MTT assay. Growth curves are shown; data is expressed relative to cell viability on day 0. *p* values were calculated using independent samples *t*-test, ^*^ *p* < 0.05, and ^***^ *p* < 0.001; (**B**) and (**C**) The plate colony formation assay was performed over 14 days, initially 1000 cells were seeded per well. The numbers of colonies formed were shown; three independent experiments were performed and *P* values were calculated using the independent samples *t*-test; (**D**) and (**E**) Cell cycle analysis of HUVEC-mock, HUVEC*-PGI2S* and HUVEC-*PGI2S-tPA* cell lines, and the percentage of cells at different phases of the cell cycle.

The result from the MTT assay showed that the proliferation of HUVEC-*PGI2S* cells was significantly reduced compared to the negative control HUVEC-mock cells, and the proliferation of HUVEC-*PGI2S-tPA* cells was even slower than that of HUVEC-*PGI2S* cells (Figure 2A). Figure 2B,C shows that the colony formation ability of both HUVEC-*PGI2S* and HUVEC-*PGI2S-tPA* cells was also reduced compared to HUVEC-mock cells. Flow cytometric examination of the cell cycle indicated G1 phase arrest occurred in both HUVEC-*PGI2S* and HUVEC-*PGI2S-tPA* cells; the extent of G1 phase arrest was more significant in HUVEC-*PGI2S-tPA* cells (Figure 2D,E). These results indicate that both the proliferation and growth ability of HUVEC-*PGI2S* and HUVEC-*PGI2S-tPA* cells were reduced in comparison to HUVEC-mock cells.

### 2.3. HUVEC-PGI2S and HUVEC-PGI2S-tPA Cell Lines Expressed Higher Levels of Anticoagulation Factors

**Figure 3.**
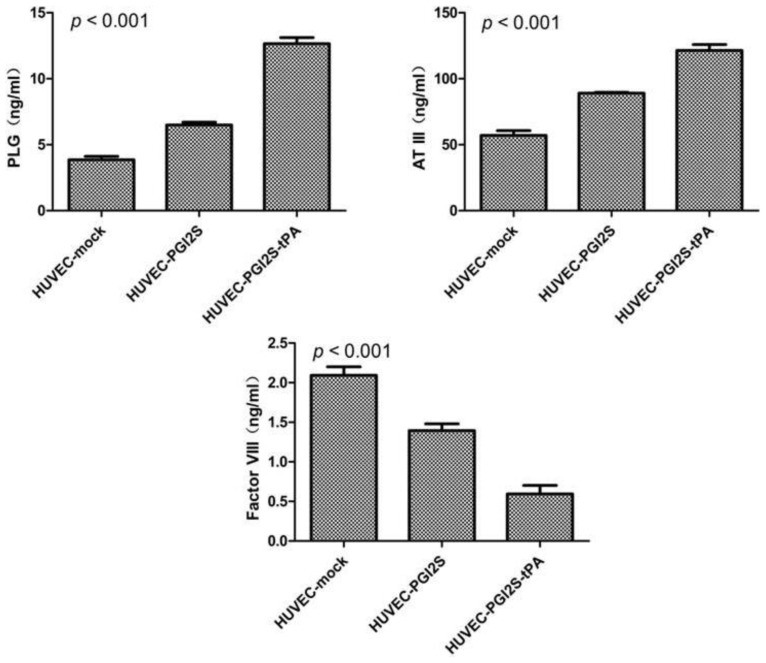
Expression of anticoagulation factors by HUVEC-mock, HUVEC-*PGI2S*, and HUVEC-*PGI2S-tPA* cells, ELISA were performed to measure the levels of *ATIII*, *PLG* and *Factor VIII* in cell homogenates, *p* values were calculated using one-way ANOVA.

The protein expression levels of *ATIII*, *PLG* and *FVIII* were examined using ELISA assays (Figure 3). *ATIII* acts as an inhibitor of several coagulation factors, and its expression was up-regulated from 57 ng/mL in HUVEC-mock cells to 89 ng/mL in HUVEC-*PGI2S* cells and 121 ng/mL in HUVEC-*PGI2S-tPA* cells (*p* < 0.001). *PLG* plays a vital role in fibrinolysis, and, *in vivo*, is activated by *tPA* in plasma. The expression of *PLG* was elevated by 2-fold and 4-fold in HUVEC-*PGI2S* and HUVEC-*PGI2S-tPA* cells, respectively, compared to the negative control cells (*p* < 0.001). On the other hand, *FVIII*, a participant in both the intrinsic and extrinsic coagulation pathways, was down-regulated by 45% and 70% in HUVEC-*PGI2S* and HUVEC-*PGI2S-tPA* cells compared to the negative control cells, respectively (*p* < 0.001).

### 2.4. Signaling Pathways Involved in Vasodilation and Platelet Disaggregation Are Activated in HUVEC-PGI2S and HUVEC-PGI2S-tPA Cell Lines

**Figure 4.**
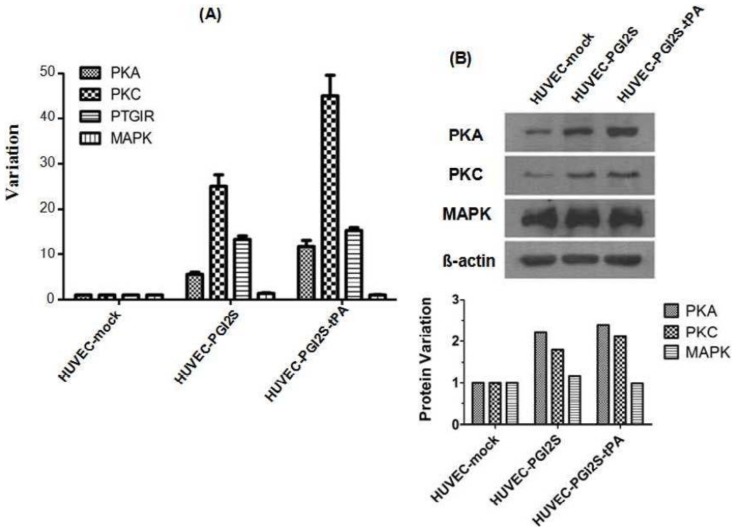
Vasodilation and platelet disaggregation signaling pathway factors in HUVEC-mock, HUVEC-*PGI2S* and HUVEC-*PGI2S-tPA* cell lines. (**A**) Analysis of *PKA*, *PKC*, *PTGIR* and *MAPK* mRNA expression by qRT-PCR; (**B**) Analysis of PKA, PKC and MAPK protein expression by Western blotting. All analysis were performed triplicated.

*PGI2*, whose synthesis is catalyzed by *PGI2S*, combines with the *PTGIR* to activate *PKA*, resulting in vasodilation and platelet disaggregation. The *PKC* pathway acts upstream of *PGI2S*, and *PKC* can also activate platelets via other signaling proteins, including *MAPK. PKA*, *PKC*, *PTGIR* and *MAPK* mRNA expression were analyzed by qRT-PCR. The expression levels of all of these signaling molecules, except for *MAPK*, were up-regulated in HUVEC-*PGI2S* cells compared to HUVEC-mock cells, and even higher in HUVEC-*PGI2S-tPA* cells (Figure 4A). Up-regulation of PKA, PKC except for MAPK protein expression was confirmed by Western blotting (Figure 4B). These results indicate that over-expression of *PGI2S* improves the anticoagulant effect of HUVEC cells, and that these effects were more potent when *PGI2S* was over-expressed in combination with *tPA*.

## 3. Discussion

CABG is an effective therapy for coronary artery disease. However, the conduits used for the bypass play an important role in the success of CABG. The use of heterograft vessels constructed from endothelial cells with improved anticoagulant ability represents a promising area of research in CABG. HUVEC cells are reported to have a relatively low immunogenicity [21,22], suggesting that transgenic HUVEC cells may be the ideal epithelial cells for developing heterograft vessels. The data presented in this study indicates that our novel transgenic HUVEC cell lines, HUVEC-*PGI2S* and HUVEC-*PGI2S-tPA* cells, may have a more potent anticoagulant effect, which may enable their future development as cells for bypass conduits.

To explore the anticoagulant effect of HUVEC-*PGI2S* and HUVEC-*PGI2S-tPA* cells, we examined their expression of vascular endothelial cell anticoagulant and coagulation factors using ELISAs. *ATIII* and *PLG* are the key components of the anticoagulation and antifibrinolytic systems. *ATIII*, a serine protease inhibitor belonging to the humoral anticoagulant system, plays an integral role in the coagulation system by inhibiting thrombin and other clotting factors [23–25]. *PLG* dissolves fibrin in blood clots and is an important protease involved in a variety of other cellular processes [26,27]. Another key protein, *Factor VIII (FVIII)*, is involved in both the intrinsic and extrinsic coagulation pathways [28,29]. Our results showed that the coagulation factor inhibitors *ATIII* and *PLG* were up-regulated, and *FVIII*, the main coagulation pathway promoter, was down-regulated in both HUVEC-*PGI2S* and HUVEC-*PGI2S-tPA* cells. Therefore, HUVEC-*PGI2S* and HUVEC-*PGI2S-tPA* cells have a more potent anticoagulant effect than HUVEC cells.

Additionally, the expression of proteins involved in the signaling pathways which regulate vasodilation and platelet disaggregation were investigated. *Epithelial growth factor (EGF)*, *vascular endothelial growth factor (VEGF)*, *Bradykinin (BK)*, *epinephrine and angiotensin II* all activate the *PKC-MAPK* signaling pathway, via phosphorylation of *MAPK*, which results in increased formation of *PGI2* [30,31]. Thus, up-regulation and activation of *PKC* are key upstream events required for synthesis of *PGI2*. Our qRT-PCR and Western blotting results demonstrated that expression of *PKC* increased in both HUVEC-*PGI2S* and HUVEC-*PGI2S-tPA* cells, *PKC* has been reported to be widely expressed in various types of tissues and plays a role in cardiovascular disease. The expression of *MAPK* remained unchanged in both HUVEC-*PGI2S* and HUVEC-*PGI2S-tPA* cells compared to HUVEC-mock cells, perhaps reflecting the fact that activation of *MAPK* may only require it to be phosphorylated rather than over-expressed.

Generation of cAMP and activation of *PKA* also form part of another important signaling pathway which regulates the biological functions of *PTGIR* activation, which in the presence of *PGI2*, results in vasodilation and inhibition of platelet aggregation [32]. In this study, *PKA* was observed to be over-expressed in both HUVEC-*PGI2S* and HUVEC-*PGI2S-tPA* cells, implying activation of this signaling pathway. Furthermore, *PTGIR* was also found to be over-expressed in both cell lines, indicating an increased sensitivity to *PGI2*. A recent study indicated that activation of *PTGIR* could protect the vascular epithelium from arteriosclerosis obliterans, the condition in which vascular grafts develop atherosclerosis a few years after CABG [33]. We hypothesize that grafts constructed from HUVEC-*PGI2S* and HUVEC-*PGI2S-tPA* cells may have a lower risk of developing atherosclerosis.

However, the results of the cell viability assay and colony formation assay showed that HUVEC*-PGI2S* and HUVEC-*PGI2S-tPA* cells are not currently fit for clinical use. Over-expression of *PGI2S* and *tPA* in HUVEC cells reduced their proliferation and growth ability. Cell cycle assays indicated that the limited cell growth and proliferation in HUVEC-*PGI2S* and HUVEC-*PGI2S-tPA* cells were due to increased G1 phase arrest. The mechanisms involved in this process are not clear, and further study is needed. Additionally, increased cell proliferation ability would be required in order for these transgenic endothelial cells to be used in clinical applications.

## 4. Experimental Section

### 4.1. Cell Culture

All HUVEC cells were cultured in RPMI-1640 medium (Life Technologies, Carlsbad, CA, USA) supplemented with 10% fetal bovine serum (FBS; Thermo Fisher Scientific, Waltham, MA, USA). HUVEC cells were stably transfected with *PGI2S*, or *PGI2S* and *tPA*, to establish the HUVEC-*PGI2S* and HUVEC*-PGI2S-tPA* cell lines, respectively. HUVEC cells were also stably transfected with lentivirus packed with pSin-EF2-puro-oligo to generate the HUVEC-mock negative control cell line. After infection, HUVEC-mock HUVEC-*PGI2S*, and HUVEC-*PGI2S-tPA* cells were treated with puromycin for four weeks to select for stably-expressing cells. Expression of P*GI2S* and *tPA* was confirmed after the four-week selection process before the other experiments were performed.

### 4.2. Plasmid Construction

Primers specific for *PGI2S* cDNA incorporating *Bam*HI and *Eco*RI restriction sites (forward: 5′-CGGGATCCATGCTCGCCCGCGCCCTGCT-3′ and reverse: 5′-CGGAATTCCTACAGTTCAGT CGAACGTTCTT-3′) and, primers specific for *tPA* cDNA incorporating with *Bam*HI and *Spe*I restriction sites (forward: 5′-CGGGATCCATGGATGCAATGAAGAGAGGGCT-3′ and reverse: 5′-GGACTAGTTCACGGTCGCATGTTGTCAC-3′) were designed and constructed. PCR amplifications of the *PGI2S* and *tPA* cDNAs were performed with the specific primers using PrimeSTAR HS (TAKARA, Otsu, Japan) using human liver cell line L-02 (Chinese Academy of Sciences, Shanghai, China) cDNA as the template. The purified PCR products and pSin-EF2-puro-oligo plasmid (James Thomson Lab, Madison, WI, USA) were digested with the corresponding enzymes, and then ligated.

### 4.3. RNA Preparation and Quantitative Real-Time PCR (qRT-PCR)

Total RNA was extracted from HUVEC, HUVEC-*PGI2S* and HUVEC-*PGI2S*-*tPA* cells using TRIzol reagent (Life Technologies, Carlsbad, CA, USA) and reverse transcription was performed using the SuperScript First-Strand Synthesis System for RT-PCR (Life Technologies, Carlsbad, CA, USA), according to the manufacturer’s instruction. After reverse transcription, real-time-PCR was performed with Platinum SYBRGreen PCR SuperMix-UDG (Life Technologies, Carlsbad, CA, USA) using the CFX96™ Real-Time PCR Detection System and Bio-Rad CFX Manager (Bio-Rad, Hercules, CA, USA) following the manufacturer’s protocol. Briefly, 2 μg of total RNA was amplified in 20 μL reactions, containing 0.5 μL M-MLV reverse transcriptase, 0.5 μL RNasin inhibitor, 0.5 μg random hexamers, 4 μL of 5× RT buffer, and RNase free water. qRT-PCR was performed in total reaction volumes of 20 μL, containing 1 μL cDNA sample, 10 μL of 2× Platinum SYBRGreen PCR SuperMix-UDG, primers, and nuclease-free water. The three-step reaction protocol was: 95 °C for 2 min, followed by 40 cycles at 95 °C for 30 s, 60 °C for 35 s and 72 °C for 30 s. *β-actin* mRNA was amplified as an internal control. The primers used were as follows: *β-actin*, forward 5′-TGGCAC CCAGCACAATGAA-3′ and reverse 5′-CTAAGTCATAGTCCGCCTAGAAGCA-3′; *PGI2S*, forward 5′-TTGCTGGCAGGGTTGCTGGTGGTA-3′ and reverse 5′-AAGATGGCATCTGGCCGAGGCTT3′; *tPA*, forward 5′-AAGCATGAGGCCTTGTCTCCTT-3′ and reverse 5′-TGCGGCCATCGTTCAGACA C-3′; protein kinase A (*PKA*), 5′-CGGAAGGTTCAGTGAGCCCCATGC-3′ and reverse 5′-GCAGATT CTCCGGCTTCAGGTC-3′; protein kinase C-alpha (*PKC*), forward 5′-TTATGCTGTCATGTCCCG GAACAG-3′ and reverse 5′-AGGAGGCGATGATGATGAGGAC-3′; *PGI2* Receptor (*PTGIR*), forward 5′-CAGAGCTTGAGTCGCTGGAA-3′ and reverse 5′-CCTCTCACGATCCGCTGCTT-3′; mitogen-activated protein kinase (*MAPK*), forward 5′-GGCATGGTGTGCTCTGCTTA-3′ and reverse 5′-CGATGGTTGGTGCTCGAATA-3′.

### 4.4. Western Blotting

Protein samples were extracted from HUVEC, HUVEC-*PGI2S* and HUVEC-*PGI2S-tPA* cells using ProteoJET Mammalian Cell Lysis Reagent (Thermo Fisher Scientific, Waltham, MA, USA) according to the manufacturer’s instruction, mixed with DualColor Protein Loading Buffer (Thermo Fisher Scientific, Waltham, MA, USA) and boiled for 5 min. Sodium dodecyl sulfate-polyacrylamide gel electrophoresis (SDS-PAGE) was performed on 4% spacer and 10% separation gels; 30 μg protein was loaded into each well. Afterwards, the proteins were transferred to polyvinylidene fluoride (PVDF) membranes (EMD Millipore, Billerica, MA, USA), blocked with 5% non-fat dry milk for 1 h at room temperature, then incubated with primary antibodies specific for *PGI2S* (52 kDa; 1:200; Santa Cruz Biotechnology, Santa Cruz, CA, USA), *tPA* (65 kDa; 1:500; Bioworld, St. Louis Park, CA, USA), *PKA* (43 kDa; 1:500; Bioworld), *PKC* (80 kDa; 1:2000; Epitomics, Burlingame, CA, USA) and *MAPK* (44 kDa; 1:1000; Cell Signaling Technology, Danvers, MA, USA) or β-actin (45 kDa; 1:1000; Cell Signaling Technology) for 12 h at 4 °C. Membranes were incubated with secondary antibodies labeled with horseradish peroxidase for 1 h, imaged using an ECL system (Pierce, Madison, WI, USA) and densitometric analyses was performed by normalization against the internal loading control β-actin using Image J software (NIH, Bethesda, MD, USA).

### 4.5. Cell Proliferation Assays

Growing cells were seeded into 96-well plates (5 × 10^3^ cells in 200 μL medium per well), cultured for 0, 1, 3, 5 or 7 days, and then 20 μL of sterile 5 mg/mL 5-dimethylthiazol-2-yl-2,5-diphenyltetrazolium bromide (MTT; Sigma-Aldrich, St. Louis, MO, USA) was added per well, and the cells were incubated for 4 h at 37 °C. After removing the medium, 150 μL dimethyl sulfoxide (DMSO, Sigma-Aldrich, St. Louis, MO, USA) was added to each well, mixed thoroughly for 15 min, and the optical density (OD) values were read at 490 nm using a Bio-Rad 3500 Microplate Reader (Bio-Rad, Hercules, CA, USA). The relative numbers of cells were expressed as percentages of the readings taken on day 0. All experiments were performed in triplicate.

### 4.6. Plate Colony Formation Assays

Cells were seeded into six-well plates (1 × 10^3^ per well) and cultured for two weeks. The colonies were fixed in methanol for 10 min and stained with 1% crystal violet for 1 min. The plate colony formation assay was performed in triplicate for each group of cells.

### 4.7. Cell Cycle Analysis

Cell cycle analysis was performed using propidium iodide (PI, Sigma-Aldrich, St. Louis, MO, USA) staining. The cultured HUVEC, HUVEC-*PGI2S* and HUVEC-*PGI2S-tPA* cells were digested with 0.25% trypsin (Life Technologies, Carlsbad, CA, USA), washed in phosphate-saline buffer (PBS), fixed in 1 mL cold 70% ethanol for 1 h, washed and centrifuged. The cell pellet was resuspended in 500 μL PI solution (50 μg/mL PI, 0.1 mg/mL RNase A, 0.05% Triton X-100 in PBS) and incubated for 40 min at 37 °C, then the cells were centrifuged and resuspended in 500 μL PBS for flow cytometric analysis using the Cytomics™ FC500 Flow Cytometry (Beckman Coulter, Brea, CA, USA).

### 4.8. Enzyme-Linked Immunosorbent Assays

The cultured cells were digested, resuspended in PBS and stored overnight at −20 °C. After two freeze-thaw cycles, the cells were centrifuged, and the resulting supernatants were used as the cell homogenates. Enzyme-linked immunosorbent assays (ELISAs) were performed on the cell homogenates using the Human Coagulation *Factor VIII (FVIII)* ELISA kit (Cusabio, Wuhan, China), Human *Antithrombin III (ATIII)* ELISA kit (Cusabio) and ELISA kit for plasminogen (USCN, Wuhan, China) following the manufacturer’s protocol. Standard curves were constructed and used to determine the expression level of each molecule.

## 5. Conclusions

The development of heterograft conduits is one of the most promising new sources of CABG grafts. Although our transgenic HUVEC cells still have a number of issues preventing their use in clinical applications, this study provides strong evidence to suggest that transgenic HUVEC-*PGI2S* and HUVEC-*PGI2S-tPA* vascular endothelial cells have the potential to be developed as ideal cells for the creation of heterograft vascular conduits.
